# Crosstalk of low density lipoprotein and liposome as a paradigm for targeting of 5-fluorouracil into hepatic cells: cytotoxicity and liver deposition

**DOI:** 10.1080/21655979.2021.1896202

**Published:** 2021-03-08

**Authors:** Saleh A. Alanazi, Gamaleldin I. Harisa, Mohamed M. Badran, Fars K. Alanazi, Ehab Elzayat, Abdullah H. Alomrani, Osaid T. Al Meanazel, Ahmed T. Al Meanazel

**Affiliations:** a Kayyali Chair for Pharmaceutical Industries, Department of Pharmaceutics, College of Pharmacy, King Saud University, Riyadh, Saudi Arabia; b Pharmaceutical Care Services, King Abdulaziz Medical City, Ministry of National Guard Health Affairs, Riyadh, Saudi Arabia; c Department of Pharmaceutics, College of Pharmacy, King Saud University, Riyadh, Saudi Arabia; d Department of Biochemistry, College of Pharmacy, Al-Azhar University, Nasr City, Cairo, Egypt; e Department of Pharmaceutics, College of Pharmacy, Al-Azhar University, Nasr City Cairo, Egypt; f Nanobiotechnology Unit, Department of Pharmaceutics, College of Pharmacy, King Saud University, Riyadh, Saudi Arabia; g Michael Sayegh Faculty of Pharmacy, Aqaba University of Technology, Aqaba, Jordan; h Prince Naif for the Health Research Center, King Saud University, Riyadh, Saudi Arabia

**Keywords:** 5-fluorouracil, 5-FUC, liposomes, cytotoxicity, LDL, liver targeting

## Abstract

This study aimed to utilize cholesterol conjugation of 5-fluorouracil (5-FUC) and liposomal formulas to enhance the partitioning of 5-FU into low density lipoprotein (LDL) to target hepatocellular carcinoma (HCC). Thus, 5-FU and 5-FUCwere loaded into liposomes. Later, the direct loading and transfer of 5-FU, and 5-FUC from liposomes into LDL were attained. The preparations were characterized in terms of particle size, zeta potential, morphology, entrapment efficiency, and cytotoxicity using the HepG2 cell line. Moreover, the drug deposition into the LDL and liver tissues was investigated. The present results revealed that liposomal preparations have a nanosize range (155 − 194 nm), negative zeta potential (- 0.82 to – 16 mV), entrapment efficiency of 69% for 5-FU, and 66% for 5-FUC. Moreover, LDL particles have a nanosize range (28–49 nm), negative zeta potential (- 17 to −27 mV), and the entrapment efficiency is 11% for 5-FU and 85% for 5-FUC. Furthermore, 5-FUC loaded liposomes displayed a sustained release profile (57%) at 24 h compared to fast release (92%) of 5-FU loaded liposomes. 5-FUC and liposomal formulas enhanced the transfer of 5-FUC into LDL compared to 5-FU. 5-FUC loaded liposomes and LDL have greater cytotoxicity against HepG2 cell lines compared to 5-FU and 5-FUC solutions. Moreover, the deposition of 5-FUC in LDL (26.87ng/mg) and liver tissues (534 ng/gm tissue) was significantly increased 5-FUC liposomes compared to 5-FU (11.7 ng/g tissue) liposomal formulation. In conclusion, 5-FUC is a promising strategy for hepatic targeting of 5-FU through LDL-mediated gateway.

## Graphical abstract

**Figure:**
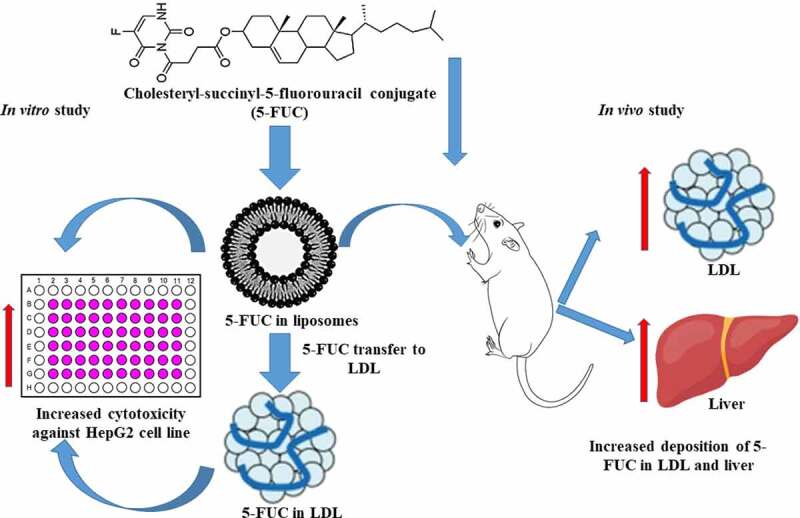

## Introduction

The liver is an essential organ responsible for many vital functions such as detoxification, protein synthesis, as well as many other biological functions [1]. Hepatocellular carcinoma (HCC) progress is caused by continuous xenobiotic exposure, chronic liver diseases, alcohol addiction, chronic viral hepatitis, fatty liver diseases as well as genetic factors [2]. HCC is life-threatening, and it is the third leading cause of cancer deaths worldwide. Chemotherapy is used clinically in HCC treatment [2]. Unfortunately, most of the chemotherapeutic agents lack specificity and induced numerous side effects [3]. Therefore, the development of hepatic-specific drug delivery systems is increasing the therapeutic index of chemotherapy. Further studies are required to address this issue.

Hepatic drug delivery is hampered by many obstacles, such as residence time in the liver and macrophages clearance of drug delivery cargoes [4]. Many approaches have been proposed to selectively deliver therapeutics to the liver [4]. Thus targeting cells-receptors is considered a novel effective approach. Low-density receptors (LDL-r) are over-expressed in many cancerous tissues including HCC. In this regard, LDL-r represents a promising tool for liver drug targeting [4–6]. Naturally, low-density lipoprotein (LDL) internalizes hepatocytes via receptor-mediated endocytosis via interaction between ApoB100 (LDL recognition protein) and LDL-r [6]. LDL pathway has been explored for targeting of many medicines into cancer cells. In this context, nanoparticles loaded with cholesterol-conjugated siRNA and doxorubicin were delivered to tumor cells through LDL gateway [7]. Besides, LDL-labeled nanoparticles loaded with another antitumor agent have been delivered by the LDL pathway [8].

5-fluorouracil (5-FU) is one of the chemotherapeutic agents used in HCC treatment. However, it has severe side-effects of 5-FU such as cardiotoxicity, myelosuppression, neurotoxicity, and other side effects [3,9]. These effects have resulted from a 5-FU water solubility drug, short half-life, decreased bioavailability, and nonspecific biodistribution [3,9]. Therefore, cholesterol conjugated of 5-fluorouracil (5-FUC) has been proposed as a prodrug for selective tumor delivery of 5-FU with improved pharmacokinetic parameters [10]. The detailed synthesis of 5- FUC was achieved by our colleagues Radwan and Alnazi, the synthesis is based on cholesterol conjugation of 5-FU to produce 5-FUC [10]. 5-FUC is a lipophilic drug, with improved plasma concentration, area under the curved, and bioavailability compared with 5-FU [11].

Theoretically, conjugation of 5-FUC mimics native cholesteryl esters as a natural component of LDL. Thus, in the physiological environment, cholesterol drug conjugates could transfer into LDL. Consequently, LDL drug cargoes traffic plasma membrane through LDL-r by receptor-mediated endocytosis. Particularly, LDL-r presents normally in the liver at a high level as well as overexpressed in HCC [6]. A previous study reported that 5-FUC shows high tumor selectivity than 5-FU [10]. Further studies are required to confirm this postulation. In this context, several published studies have developed different vehicles to facilitate drug transfer into LDL, consequently, the drug cargoes are trafficking into the tumor cells that overexpressed LDL-r [4]. Nanostructure lipid carriers containing cholesterol were concentrated in the tumor cells via the LDL-r trafficking mechanism [12]. Similarly, solid lipid nanoparticles could target LDL-r [13]. Also, nanoemulsions were successfully used for the delivery of 5-FUC conjugate to cancer cells [14]. Among the aforementioned formulations, liposomes have been shown to possess the highest drug loading ability into LDL in comparison with other carriers [15]. This might have attributed to liposomes have LDL tropism and LDL has enzymes involved in the metabolism of liposomal phospholipids.

Therefore, the main goal of the current study to use liposomal formulation as a smart vehicle to enhance the partition of 5-FUC into LDL nanocarriers. Thus, liposomes loaded with 5-FU and 5-FUC were prepared and characterized pharmaceutically. Loading efficiency of 5-FU and 5-FUC into LDL, besides the drug transfer from liposomal formulation into LDL nanoparticles were studied. The antitumor activity of formulations was studied using the HepG2 cells line as a surrogate model for HCC. Moreover, the drug deposition into LDL and liver tissues was investigated in rats.

## Materials and methods

### Material

The 5-FU and cholesterol (CHO) were purchased from Beijing Mesochem Technology CO. Ltd. (Beijing, China). 5-FUC was prepared in our laboratory, Kayyali Chair for Pharmaceutical Industries (King Saud University, Riyadh, Saudi Arabia). Dipalmitoylphosphatidylcholine (DPPC) was purchased from Avanti Polar Lipids, Inc. (Toronto, Canada). Chromatography grade methanol (MeOH) and chloroform (CHCl3) were obtained from Sigma Aldrich (St. Louis, MO, USA). All other chemicals and reagents were of analytical grade and used without any further purification.

### Preparation and characterization of 5-FU and 5-FUC liposomes

Liposomal formulations for 5-FU and 5-FUC were developed using the thin-film hydration method as reported previously [16]. For the preparation of 5-FUC loaded liposomes, CHO, DPPC, and 5-FUC were dissolved in a binary mixture of CHCl_3_ and MeOH (2:1) in a round bottom flask. The mass ratio of 5-FUC to lipid was 1:5. The mass ratio of total lipid to water was 1:38. The ratio of CHO and DPPC ratios was 0.3:0.7 molar ratio. The solvents were completely evaporated at 60°C under vacuum using Buchi Rotavapor for 1 h to form a thin lipid film on the bottom of the flask. The obtained film was hydrated at the temperature above the phase transition temperature of the lipids using phosphate buffer (pH 7.4). The hydration was performed for several hours with the help of a water bath shaker. To achieve a homogeneous dispersion of liposomes and to reduce the particle size, the liposomal suspension was sonicated for 6 min and 70% AMPL in 130-Watt Ultrasonic Processors. In 5-FU liposomal formulation, the lipids used were CHO and DPPC 5-FU was dissolved in the hydration liquid (2 mg/ml). The drug/lipid ratio and lipid: water ratio were the same. Sonication time was kept constant at 6 min. Table 1 displayed the lipids content of liposomal formulations.

**Table 1.** Particle size, zeta potential, and entrapment efficiency(EE) of the prepared liposomes

Items	Plain liposome	5-FU liposome	5-FUC liposome
Particle size (nm)	182.6 ± 26.58	193.5 ± 34.66	155.0 ± 12.24
Zeta potential (mV)	−0.82 ± 0.09	−0.73 ± 0.12	−16.2 ± 1.54
EE (%)	-	69.4 ± 0.56	66.2 ± 0.48

Data expressed as mean ± SD, N = 3

Both 5-FU and 5-FUC liposomes were characterized in terms of particle size (PS) and zeta potential (ZP) were evaluated by the dynamic light scattering method using a Zeta sizer Nano ZS (Malvern Instruments, UK). Furthermore, the liposomal formulations of both drugs were visualized using a transmission electron microscope (TEM) to evaluate their shape and morphology (JEOL JEM 1011 F, USA). The entrapment efficiency (EE %) of FU and 5-FUC in liposomes was determined by the ultra-centrifugation method. The *in vitro* release profiles of 5-FU and 5-FUC from liposomes were studied using the dialysis method [16,17].

### Preparation and characterization of LDL as drug nanocarriers

Commercial LDL nanoparticles were obtained for Sigma Louis and used to encapsulate 5-FU and 5-FUC. The biochemical components of obtained LDL particles were confirmed in the term of for cholesterol, phospholipids, triacylglycerol, and protein using commercially available kits. SDS-PAGE was performed to confirm the existence of Apoprotein B100 in LDL particles. Protein concentrations of lipoprotein segments were measured via the Markwell modification process of the Lowry protein assay method [18]. SDS-PAGE was performed according to the Laemmli method [19] using 4% gradient acrylamide gels in the Mini Protean TGX™ Precast Gels system (BioRad Laboratories, Hercules, USA). The samples were then loaded (approximately 7 μl sample per lane) on gel electrophoresis at 100 V for two hours. The gel was stained with Coomassie Brilliant Blue R-250 overnight and followed by destaining. Finally, the gel was imagined by an electrophoretic imaging system (Aplegen Omega Lum G, USA). The particle size and zeta potential were measured after dilution with phosphate buffer saline (PBS) [20] by dynamic light scattering technique using a Zeta sizer Nano ZS (Malvern Instruments, UK). Also, the morphology of LDL particles was visualized using a Transmission Electron Microscopy (TEM) to evaluate their size and morphology (JEOL JEM 1011 F, USA).

### Direct loading of 5-FU and 5-FUC into LDL

The direct addition method was used in 5-FU loading into the LDL at ratio 1:1. A 5-FUC has low water solubility, therefore, the dry film method was utilized to load 5-FUC into LDL. An aliquot of the chloroform solution of 5-FUC was dried down under a stream of nitrogen gas in a test tube. When the chloroform was completely removed, a thin dry film at the bottom of each test tube was formed. LDL was then added, and the mixture was incubated in the dark with continuous gentle shaking at 37°C for 4 h [21]. On the other hand, 5-FU is water-soluble, therefore, it is directly added to LDL with gentle shaking and incubated at 37°C for 4 h [22]. The LDL loaded with the drugs were isolated by ultracentrifugation at a density of 1.063 g/mL at 40,000 rpm for 40 h using 75 Ti Beckman rotors. The loaded LDL layer appears as a raft at the top, then aspirated and subjected to dialysis for 24 h to remove the free drug. The drug-loaded LDL nanocarriers were stored at 2 to 8°C.

### Liposomal transfer of 5-FU and 5-FUC into LDL

The liposomal transfer into LDL studies was performed as following 200 µl of liposomes containing either 5-FU or FUC was added to 200 µL of LDL solution. The mixture liquid was incubated for 2 and 4 h at 37°C under liquid nitrogen with continuous shaking. At the end of incubation, LDL particles were separated from liposomes by density gradient methods [14]. Finally, the drug partitioned into LDL particles was determined as following, 200 µL of LDL loaded with the drugs was taken in 1.5 mL centrifuge tubes. Any protein present in the samples was precipitated and cleaned by the addition of 800 µL of methanol, followed by centrifugation at 13,000 rpm at 8°C for 15 min. The supernatants were taken and transferred to HPLC vials subjected to the analysis. Percent Drug Loading = Amount of entrapped drug* 100/Total weight

### Cytotoxicity studies

The cytotoxic effect of 5-FUC loaded into LDL, 5-FUC loaded into a liposome, and 5-FUC solution in DMSO was achieved on the HepG2 cell line using the MTT assay. HepG2 cell line was obtained from American type cell culture (ATCC, Manassas, VA, USA). This method was dependent on a colorimetric reaction based on the conversion of the MTT (yellow) color to purple (formazan production). This mechanism is attributed to the effect of the tested materials on the activity of mitochondrial succinate dehydrogenase in cells. The cells were maintained in Dulbecco’s Modified Eagle Medium (Gibco, Grand Island, NY) supplemented with 10% fetal bovine serum (Gibco) at 37°C in a 5% CO_2_ humidified incubator. Cells were plated at 1 × 104 cells per well in a 96-well plate. After overnight incubation, the cells were treated with the 5-FUC solution (1 mM), 5-FUC loaded LDL and 5-FUC liposomal preparations for 3 days. 5-FU solution was utilized as a positive control. Cell viability was calculated using the following equation:

Cell viability % = Absorbance sample/Absorbance control×100

### LDL, and liver deposition study

The liver and LDL deposition of both 5-FU and 5-FUC was carried out on 12 male Wistar albino rats. The rats were maintained per national guidelines and the study protocol was approved by our Institutional Animal Ethical Committee (Ref. No.: KSU-SE-18-27). The rats were divided into 4 groups (n = 3 each) and administrated 5-FU at dose 12.5 mg/Kg, and equivalent formulations by intraperitoneal injection [23]. Group, 1 treated with a single dose of 5-FU in liposomes for 2 h. Group 2 was treated with a single dose of 5-FU in liposomes for 4 h. Group 3 received 5-FUC loaded into liposomes for 2 h. Group 4 received 5-FUC loaded into liposomes for 4 h.

Blood samples were withdrawn from the rat’s retro-orbital plexus and placed into heparin sodium-anticoagulant tubes. The plasma samples were separated by centrifugation at 3000 rpm for 5 min. LDL was separated and purified according to manufacturer procedures (LDL/VLDL and HDL purification Kit (Ultracentrifugation Free)) (MyBioSource, sunny Southern California, San Diego, USA). Afterward, the animals were euthanized with ketamine (40 mg/kg) and xylazine (2.7 mg/kg) IM.

The liver was collected, rinsed with PBS, and blotted dry on filter paper, and weighed. The tissue samples were prepared as follows: tissues after weighing were homogenized in the appropriate amount of PBS (3 mL/g of tissue) with a homogenizer on ice-water to keep the temperature under 25°C. The tissue homogenates were used for calibration after being spiked with 25 µl internal standard (Uracil,50 µg/mL) and 375 µL methanol, then vortexed for 1 min. then the samples were processed by methanol for protein precipitation. After centrifugation at 10,000 rpm for 10 min, 400 µl of the supernatant liquid was transferred to the sample vials for quantitative analysis [23,24].

### HPLC analysis of 5-FU and 5-FUC

The analysis of 5-FU was carried out at room temperature (25 ± 1°C) using Waters HPLC system (Waters, USA) equipped with 1515 LC pump, 717 autosamplers, quaternary LC-10A VP pumps, a programmable UV-visible variable-wavelength detector, SPD-10AVP column oven, an SCL 10AVP system controller (Shimadzu, Japan) and an inline vacuum degasser was used [22]. The software utilized for data analysis was Millennium (version 32). All analysis was performed using a Lichrosphere (150 mm × 4 mm) RP Nucleodur C_18_ column (Macherey Nagel, Germany) having a 5 μm particle size. The mobile phase used for this analysis was a binary mixture of methanol and water (80:20% v/v). The mobile phase has flowed with a flow rate of 1.0 mL/min with UV detection at 254 nm. The sample volume was 10 μL for each analysis. The same instrument, same chromatographic conditions were used for the analysis of 5-FUC except for the mobile phase. The mobile phase used for the analysis of 5-FUC was a binary mixture of methanol and ethyl acetate (70:30% v/v) [24].

### Statistical analysis

All data analyses were performed using GraphPad InStat software, Version 4 (GraphPad, ISI Software Inc., La Jolla, CA, USA). The results were compared using one-way ANOVA (analysis of variance) Data were expressed as mean ± SD, p-value <0.05 were used as criteria for significance

## Results and discussion

In previous studies, several approaches were proposed to target the pharmacons actively into hepatoma cells [4]. Among these approaches, LDL was chosen this the present study. LDL is bio-nano particles with a diameter range of 20–75 nm. They have a nonpolar core consisting of cholesteryl esters and triglycerides. Moreover, LDL particles have amphipathic surface consists of free cholesterol, and phospholipid monolayer wrapped by apoproteinB100 [4,6]. LDL nanoparticles act as cargoes of lipophilic materials in the blood as well as regulate the transport and metabolism of lipids and drugs [25,26]. LDL cargoes are trafficking into the intracellular environments through interaction between ApoproteinB100 and LDL-r by receptors mediated endocytosis [4,6]. It has been reported that the expression of LDL-r increased many tumors including HCC [4,6]. Moreover, the loading of cholesterol conjugates of chemotherapeutic into LDL enhanced the selective uptake of antitumor drugs into the tumor cells [4,6].

In the bloodstream, drug cholesterol conjugates could be loaded into specific lipoproteins. Particularly, such conjugates are mimicking cholesterol esters as normal components of lipoproteins [4,10,25]. LDL-loaded drug conjugates could traffic into the cell through LDL-r mediated endocytosis similar to native LDL. Particularly, LDL-r is abundant in hepatic cells as well as they are upregulated on tumor cell membranes [4,6,26,27]. Additionally, nanostructure features, biodegradability, and biocompatibility of LDL particulates inhibit their clearance by the mononuclear phagocyte system [26,27]. In this study, 5-FUC was encapsulated into liposomes and LDL as a promising approach to target 5- FU into liver tissues in a selective manner.

### Characterization of nanoliposomes

**Figure 1.**
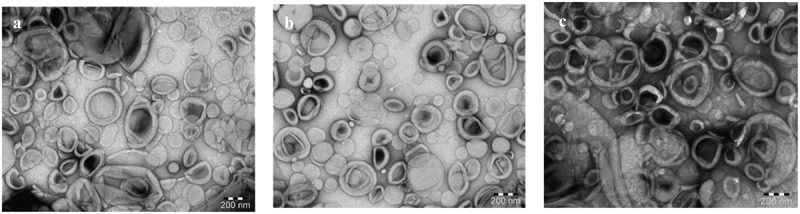
TEM images of plain liposomes (a), 5-FU loaded liposomes (b) 5-FUC loaded liposomes (c). (Bar = 200 nm)

In the present results particle size, zeta potential, and EE of the prepared liposomes were displayed in Table 1. The Data in this table showed that the prepared liposomes were in nanosize less than 200 nm. This size is desirable for the existing study, it could help in the interaction between liposomes and LDL. However, the collision of the particles increases their interaction due to a narrow particle size distribution. Moreover, the small particle size could enhance the separation of LDL from liposomal particles during the drug transfer studies. entrapment efficiency(EE) of 5-FU and 5-FUC was desirable. Additionally, In the current results, both liposomes and LDL are elicited negative zeta potential. These values were suggested to be close to the threshold of agglomeration between liposomes and LDL [28]. Additionally, TEM analysis represents the surface morphology and size distribution of optimal liposomal formula of plain liposomes, 5-FU liposomes, and 5-FUC liposomes. TEM images of liposomal formulations were illustrated in Figure 1. This figure represented that, the appearance of liposomes in vesicle shapes within the nanometer range.

**Figure 2.**
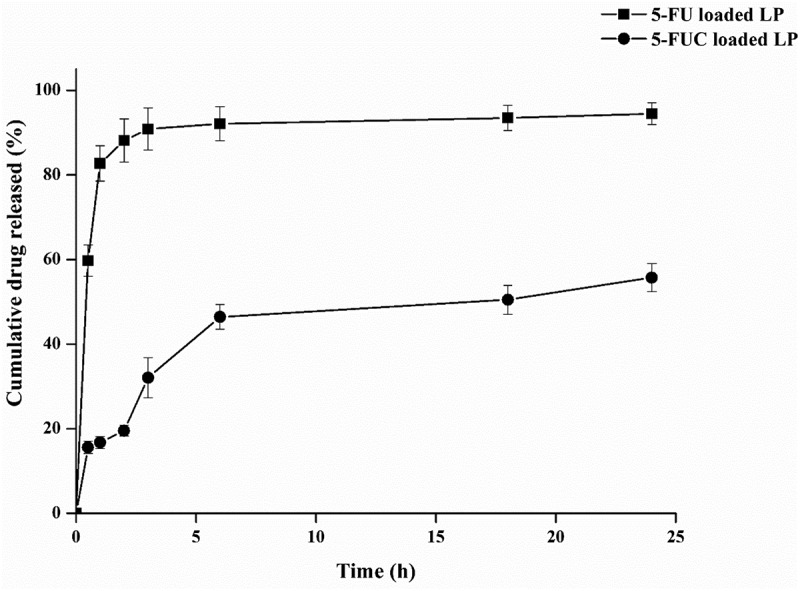
Release profiles of 5-FU and 5-FUC from liposomes. Data were expressed as mean ± SD, n = 3 in each group

*In vitro* drug releases of 5-FU and FUC from liposomes were found to reasonably fast, with an initial rapid/burst release was noted for 5-FU and 5-FUC. Also, it was noted that 5-FU had a faster release than that of 5-FUC. The present results indicated that 5-FU releases from liposomes 41% more than 5-FUC. A 5-FU is hydrophilic and easy to leak from the liposomes, while 5-FUC is hydrophobic, and its release from liposomes reaches 50% with 5 h. Figure 2 showed the drug release profiles of 5-FU and 5-FUC from liposomes. The higher release pattern of both drugs from liposomes might be advantageous during the drug transfer studies. Likewise, Kader et al. (15], studied liposomes and the physicochemical factors affecting drug loading into LDL particles. Moreover, the previous study that suggests the liposomal drug formulations could interact directly with the plasma compartments, LDL particles are one of the plasma components [29].

### Characterization of LDL as drug cargoes

**Figure 3.**
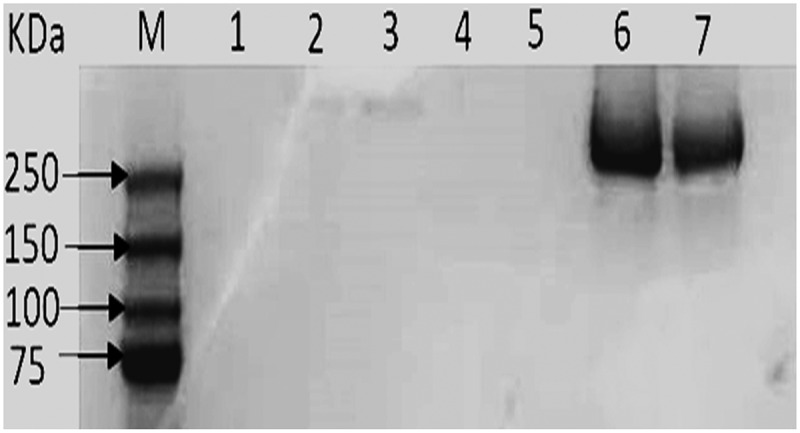
SDS polyacrylamide gel electrophoresis of the LDL. Lane M, show band corresponding to standard proteins with varying molecular weights (Da). Lane 6 and 7 were samples from separated LDL

The biochemical analysis results of LDL components in the term of for cholesterol, phospholipids, triacylglycerol, and protein revealed that observed no valuable change occurred during loading between native LDL and drug-loaded LDL. Moreover, SDS-PAGE was performed to confirm the existence of Apoprotein B100 in LDL. It was observed that, the presence of proteins with estimated molecular weight equal to 250 Kilo Dalton. Similarly, previous literature demonstrated that protein with 250 kDa is present in LDL fraction [30]. Figure 3, depicted SDS polyacrylamide gel electrophoresis of the LDL

**Figure 4.**
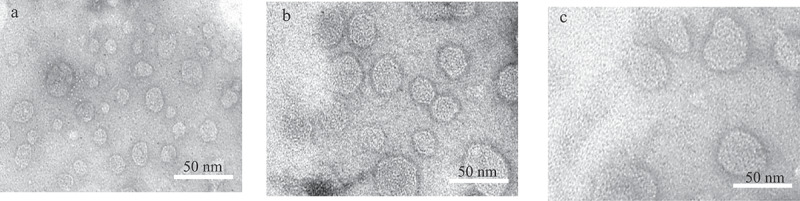
TEM images of plain LDL (a), 5-FU loaded LDL (b) 5-FUC loaded LDL (c). (Bar = 50 nm)

Table 2, depicted particle size, zeta potential, and EE of plain LDL, 5-FU-LDL, and 5-FUC-LDL. In this study particle size, and zeta potential of LDL nanoparticles were estimated using dynamic light scattering technique after dilution with PBS [20]. The particle size of LDL is found in the range of 28–48.6 nm. These results are in agreement with the finding of another study that reported the nanosize range of LDL [30,31]. In the current study, TEM images of LDL were illustrated in Figure 4. These results revealed that the LDL cargoes conserve their natural characteristics and promising for drug loading.

**Table 2.** Particle size, zeta potential, and entrapment efficiency(EE) of plain LDL as well as drugs loaded LDL

Property	Plain LDL	5-FU LDL	5-FUC LDL
Particle size (nm)	28.3 ± 4.75	31.4 ± 5.741	48.63 ± 3.85
Zeta potential (mV)	−17.1 ± 1.75	−19.2 ± 2.25	−27.0 ± 3.81
EE (%)	-	11.2 ± 1.23	84.5 ± 9.84*

Data expressed as mean ± SD, N = 3, *, significant increase at p-value <0.05

### Drug loading of drugs into LDL

**Figure 5.**
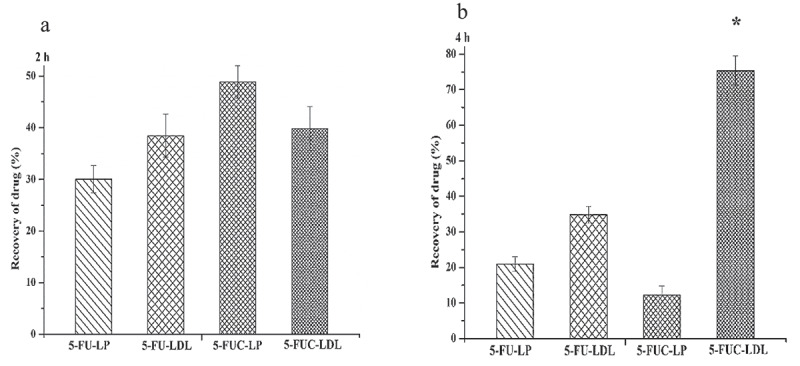
Transfer percentage of 5-FU and 5- FUC into LDL using the liposome loading method. (a) after 2 h incubation time, (b) after 4 h incubation time

There are several approaches for loading medicines into LDL. The first approach implies the substitution of the pharmacons lipid core of lipoprotein (core loading). The second is achieved through the intercalation of pharmacons into phospholipids monolayer of LDL (surface loading). The third approach ‘protein loading’ is achieved by the covalent attachment of therapeutic agents to the amino acid residues of apoproteins [6,32]. In the present study, the core and surface, approaches were utilized for loading of 5-FU and 5- FUC into LDL. The direct addition (core and surface loading), 5-FU is present in solution, while 5-FUC is present in a dry lipid layer. It was observed after 4 h incubation time, the entrapment efficiency of 5-FUC into LDL was significantly higher than 5-FU (84.5% versus 11.0%), see Table 2. These findings suggested that the hydrophobic character of 5-FUC is an important factor for LDL loading. On the contrary, 5-FU is hydrophilic with decreased loading capability into LDL. These results are in synchronization with the finding of previous studies that successfully loaded both hydrophilic and lipophilic drugs into LDL ([22]. The percentage of 5-FU and FUC transfer into LDL after 2 h and 4 h using the liposome loading method was shown in Figure 5 A and B.

Data were expressed as mean ± SD, n = 3 in each group*, significant increase at p-value <0.05

An increase of the pharmacons hydrophobicity enhance their transfer into LDL was demonstrated in numerous studies [31–33]. In this study, the drug transfer from liposomal formulas into LDL was studied after 2 h, and 4 h incubation time. After 2 h incubation time, the percent of drugs transfer was (30.1 ± 0.62), (38.3 ± .0.32), (49.3 ± 0.14), and (39.7 ± 0.32) form 5-FU liposome, 5-FU LDL, 5-FUC liposomes, and 5-FUC LDL. The present data indicated that the 5-FUC transfer was greater than that of 5-FU. The lipophilic natures of 5-FUC increase its propensity to transfer into LDL. Similarly, Kader et al. [15], demonstrated that liposomes enhance drugs into LDL particles. The present observations confirmed that conjugation of anticancer drugs with cholesterol mitigates their loading into LDL.

After 4 h of incubation, the percent of 5-FU in the liposomes and LDL layers dramatically decrease. Similarly, the percent of 5-FUC separated in liposomes dramatically decreases by 53%. Differently, the percent of 5-FUC separated in the LDL layer increases by 92.3%. This indicated that by time 5-FU moved from liposomes quickly and has trouble being loaded into LDL, while, 5-FUC molecules release from liposomes quickly and partition into LDL. These findings confirming the fact that the hydrophobic molecules have high core loading into LDL. Numerous studies reported that pharmaceutical carriers were hypothesized to enhance drug loading into LDL [12–14]. It has been reported that the liposomal formulations possess the highest ability of drug transferring into LDL [15]. The liposomes have been shown to possess the highest drug loading ability into LDL. This may be attributed to the structural similarity between liposomes and LDL. Also, LDL has enzymes used in the metabolism of phospholipids of the liposomes, both in vivo and in vitro. The present observations suggested that cholesterol conjugation of 5-FUC mitigates its loading into LDL.

Usually, the lipid-soluble materials exhibit a higher affinity for loading into LDL core [6,32]. Therefore, the loading of 5-FUC into LDL based on their intercalation within the core LDL due to mimicking native cholesterol ester. The interaction of hydrophobic agents with LDL could be performed by van der Waals forces [6,32]. While 5-FU is hydrophilic and required special transporters to internalize the cells [33]. Hence, 5-FUC move from liposomes and passively be diffused into LDL due to the hydrophobic nature.

### Cytotoxicity studies

**Figure 6.**
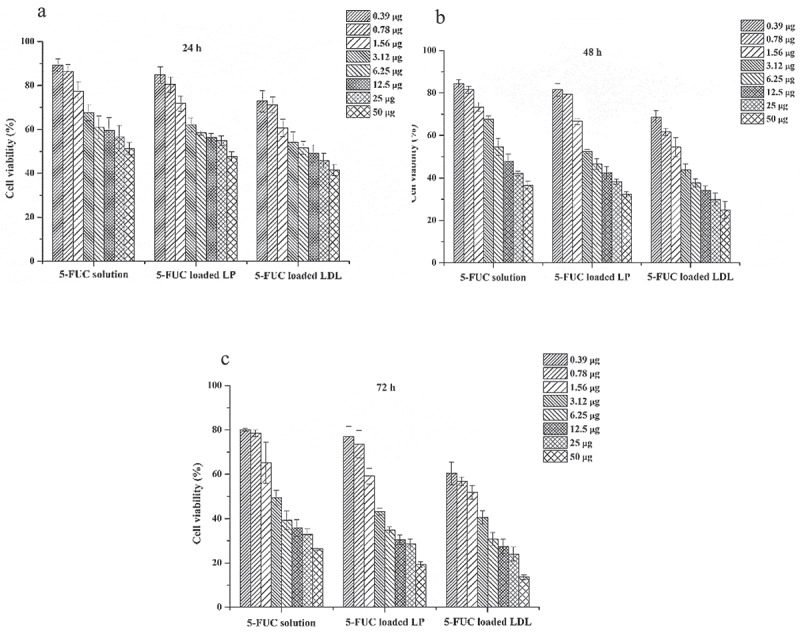
Effect of the 5-FUC solution, 5-FUC loaded liposomes, and 5-FUC loaded LDL on HepG2 line cell viability after, 24 h (a), 48 (b) 72 h (c) incubation time

In the existent work cell viability was studied for 5-FUC loaded into LDL, 5-FUC liposome, and 5-FUC solution using HepG2 cell line. The current results clear that the concentration of 5-FUC and incubation time affect HepG2 cell viability. The more concentration results in a decrease of cell viability. The highest cytotoxicity was obtained for the 5-FUC solution at 24 h and 48 h of incubation time compared to 5-FUC loaded into LDL and liposomes. Conversely, the cytotoxicity was enhanced in the group treated with drug-loaded liposomes, and LDL cargoes after 72 h time. The results of the cell viability study were illustrated in Figure 6a, B, and C. The half-maximal effective dose (IC_50_, µg/ml) was calculated to differentiate between the three delivery systems carrying 5-FUC. IC_50_ at 24 h was 10.8, 51, and 41.6 for the solution, liposomes and LDL loaded delivery system with 5-FUC, respectively. IC_50_ at 48 h was 1.6, 3.6, and 4.8 for the solution, liposomes and LDL loaded delivery system with 5-FUC, respectively. On the other hand, IC_50_ at 72 h was 1.5, 1.7, and 2.4 for the solution, liposomes, and LDL loaded delivery system with 5-FUC, respectively. These data indicated that the cytotoxicity was enhanced by drug loading into LDL. This means that 5-FUC loaded into LDL needs time to give therapeutic effect *in vitro* in comparison with the drug solution. This suggested that LDL-5-FUC loaded would enter the cell through LDLr through receptor-mediated endocytosis, and this required more time. It has been reported that 5-FU is a hydrophilic drug and traffic the plasma membrane by facilitated diffusion using a uracil transporter (33). However, 5-FUC is a lipophilic drug, hence it might traffic the cell membrane through passive diffusion and escapes lysosomal degradation. Therefore, the loading of anticancer cholesterol conjugates into liposomes or LDL is essential for tumor cell killing [22]. It is documented that, LDL cargoes are delivered into lysosomes where the degradation process has taken place [6,22]. In regards, it has been reported that lysosomal enzymes played critical roles in the drug release from nanosomes internalized into the intracellular milieu by receptors mediated endocytosis [34]. In the intracellular milieu, the lysosomal esterase converts 5-FUC to 5-FU to do the anticancer effect through inhibition of DNA replication, and transcription so inhibits the cells proliferation.

Data were expressed as mean ± SD, n = 3 in each group

The present results were similar to the previous reports stated that the prodrug design to target LDL nanoparticles is a promising tool for anticancer delivery with selective tumor targetability including HCC [4,6,35,36]. In this regard, LDL coupled nanoparticles were documented to deliver cholesterol-conjugated chemotherapeutics into hepatic cells. Furthermore, the LDL-decorated delivery system exhibited cytotoxicity against HepG2 cells [4]. As well, it has been demonstrated that LDL-based nanocarriers could enhance drug delivery to HCC cells [37]. Moreover, another study stated that bio-mantling of 5-FU loaded liposomes enhances the toxicity and targetability of 5-FU to HepG2 cell line (38). The internalization of liposomes into HepG2 cells is mediated through LDL-r that overexpressed on such cells [38].

### Deposition of the drug into LDL, and liver

In respect to LDL, and liver drug deposition, the present results revealed that the distribution of the drug in liver tissues from 5-FUC solution, liposomal, and LDL loading was significantly higher than that of 5-FU solution or in liposomal formulas, see Table 3. The cholesterol conjugation, liposomal formulation, and LDL loading enhanced the LDL, and liver deposition of 5-FU. This may be attributed to the partitioning of 5-FUC into LDL in the blood, subsequently, LDL cargoes are traveling to the hepatic tissues. In this regard, it has been reported that the increased lipophilicity of drugs by the addition of lipid moiety could improve the transfer of the drug into lipoprotein. The liver the major location for lipoprotein metabolism, herein the hepatic uptake of the chemotherapeutic agents is enhanced lipoprotein mediated gateway [36,37]. This indicated our approach could be the successful strategy of 5-FU targeting liver tissues selectively. These observations are concurrent with several previous studies that documented the liver biodistribution of drugs through LDL gateway [39]. Likewise, another study demonstrated that liposomal vehicle enhances the hepatic deposition of the drugs [39]. Table 3, depicted the biodistribution of drugs (ng/gm) from different formulations in the LDL, and liver tissues after 2 h and 4 h of intraperitoneal injection.

**Table 3.** Deposition of drugs from 5-FU and 5-FUC liposomes into LDL, and liver tissues after 2 h and 4 h of drug intraperitoneal injection

	5-FU liposomes		5-FUC liposomes	
Items	2 h	4 h	2 h	4 h
Liver tissues(ng/gm)	14.83 ± 8.21	11.7 ± 4.00	481 ± 110*	534 ± 122*
LDL (ng/mg)	NA	NA	22.24 ± 1.98	26.87 ± 2.57

Data expressed as mean ± SD, N = 3, *, significant increase at p-value <0.05

Although many studies utilizing LDL as drug delivery vehicles, their use in the clinic is still limited due to difficulty in isolation of such cargoes in vitro loading of therapeutic agents. Moreover, the samples vary from batch to batch, and it is difficult to obtain large quantities of LDL as drug cargoes [31]. Also, the difficulty of preserving lipoproteins in intact form, the potential pathogen contamination, as well as the limited stability of the resulting LDL is stilling challenges for utilization of LDL as drug delivery vehicles [40]. Most of these problems can be simplified by the *in vivo* loading of antitumor agents into native lipoproteins without extracting them from the body [41]. This approach might represent a flexible strategy to load the desired therapeutic agent into natural LDL *in vivo* by the administration of healthy fats, as well as by the induction of transient hyperlipidemia [31,40–43]. Moreover, the fabrication of LDL-like nanocarriers could be used for the same purpose [31,40–43]. These approaches might overcome the drawbacks of in vitro processing lipoproteins as drug delivery systems.

## Conclusion

This study concluded that 5-FU in the form of 5-FUC was successfully loaded into LDL as drug cargoes. This effect is due to the presence of cholesterol moiety, which drives 5-FU (hydrophilic drugs) into 5-FUC (hydrophobic drug). Herein, 5-FUC could be accumulated inside the LDL. Subsequently, drug-loaded LDL could interact with LDL-r and accumulate into cancer cells that overexpressed LDL-r with enhanced the cytotoxic effect of 5-FU against the HepG2 cell line. Moreover, the deposition of 5-FUC into LDL and liver was significantly higher than 5-FU. Furthermore, *in vivo* studies are planned to address this issue using the animal model for HCC.
